# Exploring the Relationship between Crassulacean Acid Metabolism (CAM) and Mineral Nutrition with a Special Focus on Nitrogen

**DOI:** 10.3390/ijms20184363

**Published:** 2019-09-05

**Authors:** Paula Natália Pereira, John C. Cushman

**Affiliations:** Department of Biochemistry and Molecular Biology, University of Nevada, Reno, NV 89557, USA

**Keywords:** ammonium, crassulacean acid metabolism (CAM), nitrate, nitrogen, nutrient availability, organic nitrogen sources

## Abstract

Crassulacean acid metabolism (CAM) is characterized by nocturnal CO_2_ uptake and concentration, reduced photorespiration, and increased water-use efficiency (WUE) when compared to C_3_ and C_4_ plants. Plants can perform different types of CAM and the magnitude and duration of CAM expression can change based upon several abiotic conditions, including nutrient availability. Here, we summarize the abiotic factors that are associated with an increase in CAM expression with an emphasis on the relationship between CAM photosynthesis and nutrient availability, with particular focus on nitrogen, phosphorus, potassium, and calcium. Additionally, we examine nitrogen uptake and assimilation as this macronutrient has received the greatest amount of attention in studies using CAM species. We also discuss the preference of CAM species for different organic and inorganic sources of nitrogen, including nitrate, ammonium, glutamine, and urea. Lastly, we make recommendations for future research areas to better understand the relationship between macronutrients and CAM and how their interaction might improve nutrient and water-use efficiency in order to increase the growth and yield of CAM plants, especially CAM crops that may become increasingly important as global climate change continues.

## 1. Introduction

Crassulacean acid metabolism (CAM) is characterized by nocturnal CO_2_ assimilation by phospho*enol*pyruvate carboxylase (PEPC) and nocturnal organic acid accumulation, mainly malate, into the vacuole. Nocturnal CO_2_ uptake occurs due to nighttime stomata opening, which also improves water-use efficiency (WUE), because air:leaf water vapor pressure deficits are lower at night when compared with the daytime [1]. During the day, organic acids accumulated overnight in the vacuole are decarboxylated by either the phospho*enol*pyruvate carboxykinase enzyme (PEPCK) or the NAD(P)-malic enzyme (ME), depending on the families, in order to release pyruvate and CO_2_. Pyruvate is converted to phospho*enol*pyruvate (PEP), which is the substrate for PEPC, and CO_2_ is assimilated during the day by ribulose-1,5-bisphosphate carboxylase/oxygenase (RUBISCO) [2,3,4]. The diurnal“CO_2_ pump” from C_4_ acid decarboxylation favors the carboxylase instead of the oxygenase activity of RUBISCO, which reduces photorespiration and consequently increases the efficiency of photosynthesis in CAM plants [4,5]. CAM plants can perform different degrees of CAM photosynthesis and are classified as obligate CAM, facultative CAM, CAM cycling, and CAM idling [6,7,8]. Obligate CAM plants mainly perform CAM independent of proximate environmental conditions [9,10]. Facultative CAM species perform C_3_ photosynthesis under nonstressful environmental conditions, whereas under stressful conditions such as elevated atmospheric CO_2_ concentrations, salinity, water deficit, nutrient availability, light, and temperature regimes, they switch to CAM [11,12,13,14,15]. CAM cycling is considered a very weak type of CAM that is characterized by diurnal CO_2_ uptake and slight nocturnal C_4_ acids accumulation [16,17,18]. CAM idling is characterized by nonatmospheric CO_2_ fixation wherein the stomata are closed 24 h a day and respiratory CO_2_ is refixed [19]. The induction of CAM by abiotic stresses such as water deficit, light intensity, temperature, and salinity has been well documented. In contrast, the impacts of nutritional availability on CAM have not been well explored relative to other abiotic conditions. Due to the lack of information regarding the relationship between mineral nutrients and CAM, we discuss how nutrients can modulate the CAM pathway, which mineral nutrients seem to be more important for increasing or decreasing CAM expression, the metabolic costs and benefits of inorganic and organic sources of nitrogen, which is the most limiting macronutrient for plant growth, and how this nutrient is related to the performance of CAM.

### 1.1. Induction and Regulation of CAM Photosynthesis

The plasticity of CAM has been studied in facultative CAM species that are capable of performing two photosynthetic pathways depending upon environmental conditions [15,20,21]. Much interest has been given to the induction of CAM by specific abiotic factors such as water deficit and salinity and its regulation by phytohormones such as abscisic acid (ABA) and cytokinins [14,21,22,23,24]. For example, when *Portulacaria afra* plants were exposed to water deficit conditions, stomata tended to close during the day, nocturnal acid accumulation occurred, and CO_2_ was mostly taken up at night. All of these characteristics were associated with CAM induction [22]. In this same study, Ting [22] applied ABA in nonstressed *P. afra* plants, resulting in stomata closure during the day and nocturnal CO_2_ fixation. In *Mesembryanthemum crystallinum*, leaf dehydration and ABA treatments resulted in a stronger increase in *Ppc1* transcripts, which encode a CAM-specific isoform of PEPC, compared to the control [25,26]. Another study showed that when *M. crystallinum* plants were kept under salt stress conditions, there was an increase in endogenous ABA [27]. This response was probably the result of the reduction of water content in the plants when they were kept under salt stress, which induces ABA accumulation and CAM photosynthesis [25,26]. Studies performed on *Kalanchoë blossfeldiana* showed that ABA content increased before CAM induction [24]. Besides examining gene expression and growth regulator changes to water deficit in CAM plants, biochemical approaches have been performed in order to better understand the relationship between CAM and water deficit. Ceusters et al. [28] reported on changes in different storage carbohydrate pools involved in the acclimation to water deficit and recovery from dehydration in the bromeliad *Aechmea* ‘Maya’. When *Aechmea* ‘Maya’ plants were kept under well-watered conditions, sucrose degradation accounted for only 27% of nocturnal carbohydrate supply to the provision of PEP, while starch accounted for 67%. On the other hand, after 180 days of water deficit, sucrose degradation accounted for 90% of the provision of PEP and starch accounted for only 25% [28]. The preference for sucrose over starch under prolonged water deficit conditions may be favored by energetic costs, because there are lower energy costs in terms of ATP by using soluble sugar over starch to restore PEP in CAM plants [28].

Most studies regarding CAM induction/regulation have focused on water limitation and salinity, while mineral nutrients, which are essential elements for plant growth and resistance under stressful environmental conditions and are strongly linked to water uptake, have not been well explored in CAM plants. Much more attention needs to be given to the relationship between mineral nutrients, CAM, and plant growth. The environmental productivity index (EPI), which considers water, temperature, and light indices in order to predict the yield of plant species under different environmental conditions, does not take into consideration a nutritional index parameter [29,30]. Nutrients are as important to plant yield as light, water, and temperature as different nutritional conditions can directly affect plant growth and productivity [31,32]. A better understanding of the interaction between CAM and mineral nutrient availability is needed in order to build more precise EPI models as well as to improve the growth and productivity of CAM plants. In addition to developing more precise EPI models, the understanding of mineral nutrient-use efficiency in CAM plants would be an important aspect to consider when engineering CAM into C_3_ plants in order to increase nutrient-use efficiency in combination with water-use efficiency, which will be a potentially useful advantage for C_3_ crops under the conditions of global climate change.

### 1.2. Nutrient Availability Interactions with CAM 

Nitrogen (N), potassium (K), and phosphorus (P) are essential nutrients for plant development and growth [33]. However, how these macronutrients affect CAM remains largely unclear. Besides N, P, and K, the influence of cytosolic calcium (Ca^2+^) on CAM induction has been demonstrated in *M. crystallinum* plants [25]. *M. crystallinum* detached leaves treated with 5 mM EGTA, a chelator of extracellular Ca^2+^, blocked *Ppc1*, NAD-glyceraldehyde-3-phosphate dehydrogenase (*GapC1*), and cytosolic NAD-malate dehydrogenase (*Mdh1*) transcript accumulation. When detached leaves were pretreated with ionomycin, a calcium ionophore, *Ppc1* transcript accumulation increased. These results showed that changes in Ca^2+^ cytosolic possibly act as a signaling molecule leading to CAM induction [25]. However, more studies have to be done in order to better understand how the absence/presence of calcium as a macronutrient in solutions would interfere with the CAM pathway. In *Medicago sativa*, a C_3_ species, the absence of K^+^ caused a decrease in photosynthesis and increase in stomatal resistance when compared with 4.8 mM K^+^ [34]. Although the influence of K^+^ on C_3_ photosynthesis has been studied, little information is available on how this macronutrient would independently modulate CAM expression. 

Phosphorus is a component of several molecules, nucleic acids, ATP, PPi, and phospholipids. After N, phosphorus is the second most limiting macronutrient for plant growth [35]. Several studies performed on C_3_ and C_4_ plants showed that P deficiency decreases the rate of net CO_2_ assimilation, which might result from the decrease in atmospheric CO_2_ conductance to the chloroplasts and/or from harmful effects on photosynthetic mechanisms [36]. Seedlings of *Clusia minor*, a C_3_-CAM species, cultivated under P deficiency + water deficit grew less and showed higher nocturnal organic acid accumulation compared to seedlings grown in the presence of phosphorus [36]. *Clusia minor* seedlings showed increased biomass, P, and N content, but exhibited decreased CAM expression under P fertilization. The lower N content possibly caused by P deficiency might be responsible for increasing CAM photosynthesis because N deficiency reduces growth by reducing photosynthetic rates and chlorophyll content [37,38]. *M. crystallinum* kept under nitrate and phosphate deficiencies exhibited enhanced CAM activity as a result of a reduction in stomatal conductance [39]. When phosphate deficiency was tested alongside salt stress, a higher nocturnal malate accumulation was also observed. The authors suggested that CAM induction may be a result of reduced water potential due to dehydration and increased ion concentrations under salt treatment. In addition, less growth and accumulation of pinitol under nitrate and phosphate deficiencies, which could participate in osmotic adjustment in response to the accumulation of solutes, could increase CAM [39,40]. However, more research is necessary to better elucidate the relationship between N and P deficiencies and CAM induction. In general, experiments need to be done with plants that perform different degrees of CAM such as CAM cycling, facultative and obligate CAM, and even CAM idling. Solutions should be prepared with the absence of Ca^2+^, P, N, or K in order to evaluate the influence that each macronutrient might have on the mode or magnitude of CAM being performed.

Winter and Holtum showed the effect of KNO_3_ fertilization on 24 h CO_2_ exchange in *Calandrinia polyandra* plants kept under well-watered conditions [13]. Before KNO_3_ fertilization, a slight nocturnal CO_2_ uptake was observed, while after the soil had been flushed with 20 mm KNO_3_ for two days, nocturnal CO_2_ uptake was completely lost, and the plants only assimilated atmospheric CO_2_ during the daytime [13]. This was the first study that found that soil nutrient supply affects the balance between C_3_ photosynthesis and CAM. However, further research needs to be done in order to understand which macronutrient (K^+^ or NO_3_^−^) is responsible for affecting the photosynthetic balance in *C. polyandra* [13], because both macronutrients were provided at the same time to the plants. 

**Figure 1:**
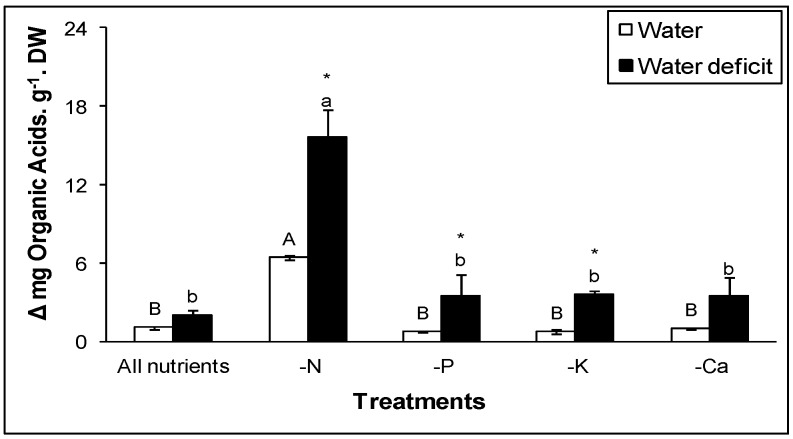
Nocturnal organic acid accumulation in the apical portion of the leaves of *Guzmania monostachia* kept under water deficit or water plus nutritional deficiency (NO_3_^−^ and NH_4_^+^, PO_4_^2−^, K, or Ca^2+^) for seven days. Data are expressed as the mean (±SE) of three replicate samples. Different capital letters indicate values that were significantly different among species using the same anion (Tukey–Kramer test; *P* < 0.05). Different lower-case letters indicate values that were significantly different among different carboxylate anions in the same species (Tukey–Kramer test; *P* < 0.05).

In detached leaves of *Guzmania monostachia*, a C_3_-CAM species, no significant difference was observed in nocturnal organic acid accumulation in the apical portion of the leaves when they were kept under the absence of Ca^2+^, PO_4_^2−^, or K^+^ plus water deficit (Figure 1). However, when the leaves were kept under the absence of only N, a five-fold increase in nocturnal organic acid was observed in the apical portion of the leaf of this bromeliad. In addition, PEPC and MDH activities were higher in the apex of the leaves kept under the absence of N plus water deficiency compared to the absence of Ca^2+^, PO_4_^2−^, or K^+^ plus water deficit [41]. Therefore, for *G. monostachia*, the absence of N in association with water deficit exhibits the strongest CAM expression in the apical portion of the leaves. A larger number of studies involving CAM induction and N have been completed compared to the research done on CAM induction and potassium or phosphorus. For this reason, the next sections will focus on nitrogen sources and CAM induction in facultative and obligate CAM species.

## 2. Relationship between Nitrogen and CAM 

A study performed on *Kalanchoë lateritia* showed not only that the presence/absence of N was important to decrease/increase CAM expression, but also that the final concentration of this macronutrient had a marked interference on CAM [42]. Researchers observed the highest PEPC activity, *Ppc1* transcript abundance, nocturnal organic acid accumulation, and nocturnal CO_2_ exchange when plants were kept under N/5 (3.1 mM) compared to N (15.5 mM) and N/10 (1.55 mM). The lowest N concentration (N/10) showed the lowest CAM expression/activity, which could be a result of an inadequate level of nitrogen, which increased the rate of senescence for these plants [42]. These results showed that for *K. lateritia*, there is an optimal N concentration that increases CAM photosynthesis. Moreover, other concentrations above or below this optimal level had a negative interference on the magnitude of CAM performance. In *Kalanchoë pinnata*, lower nitrogen concentrations (0.6 mM) were shown to decrease PEPC activity, while higher concentrations (24 mM) increased PEPC activity in mature leaves [43]. In *Coryphantha vivipara*, *Opuntia bigelovii* and *Trichocereus chilensis* (Cactaceae), lower N concentrations in the hydroponic conditions led to lower nocturnal organic acid accumulation [44]. *K. blossfeldiana* plants that were kept under the presence of 5 mM NaNO_3_ or 2.5 mM (NH_4_)_2_SO_4_ for 1–2 months, and then transferred to N-deficient conditions, showed a higher nocturnal CO_2_ uptake in the absence of either N sources when compared to the presence of NO_3_^−^ or NH_4_^+^ [45]. In addition, PEPC activity and nocturnal organic acid accumulation were higher under N deficiency. The lack of N was thus proposed to be an important factor for the stimulation of CAM in *K. blossfeldiana* [45]. In studies where *M. crystallinum*, a facultative CAM species, and *Bromus mollis*, a C_3_ species, were grown together under water-deficit or salt stress conditions, a decrease of leaf water potential of *B. mollis* was observed under high N concentrations when compared with lower N concentrations [46,47]. This response probably occurred because under high levels of nutrients, there is an increase in biomass accumulation, which leads to higher evapotranspiration and consequently lower photosynthetic rates. In contrast, *M. crystallinum* plants kept under water-deficiency or salinity stress (CAM-induced) did not show any difference in CAM expression under low or high N concentrations, probably because under low N levels, sufficient carbohydrates were provided for nocturnal carboxylation. Under water deficit, *M crystallinum* seems to be a stronger competitor than *B. mollis* because of its higher leaf water potential and photosynthetic assimilation rates. Under well-watered conditions, much lower water potential, photosynthetic rate, and total biomass were observed in *M. crystallinum*. The strong competition of *B. mollis* with *M. crystallinum* under conditions of abundant water availability might be a result of the delay in plant maturity and prevention of carbohydrate accumulation required for CAM expression in *M. crystallinum* [46,47]. These results show that species–species relationships are as important as environmental interactions with plant species in predicting which species are going to prevail under specific abiotic stresses [46]. In the future, due to global climate changes and consequently increased episodes and duration of droughts and increased nitrogen deposition, CAM species are likely to increase in dry lands. While CAM plants can maintain high photosynthetic rates under water-deficit conditions independent of N levels, C_3_ plants not only increase evapotranspiration, but also decrease photosynthetic rates. For this reason, obligate and facultative CAM species are likely to be stronger competitors and become more predominant in dry lands where soils are poor in nutrients and water. Despite several studies which have reported the influence of N on CAM photosynthesis, only recent studies have focused on understanding how this mineral nutrient interferes with CAM photosynthesis. Clearly, N source as well as its concentration can have a marked effect on the magnitude of CAM in obligate CAM species [44] or can affect the induction of CAM photosynthesis in facultative CAM plants [42,43,45,46]; however, it remains unclear if this influence is on a biochemical, molecular, and/or anatomical level and how each N source differentially influences CAM expression. In the next sections, we will explore how different nitrogen sources are transported into cells and assimilated, as well as CAM plant preferences for different N sources.

### 2.1. Nitrate, Ammonium, and Urea Uptake and Transport into the Cells

Nitrogen is a constituent of amino acids and it plays a role in almost all metabolic processes in plants [48]. Atmospheric N_2_ can be biologically reduced to ammonium by rhizobia symbioses and it accounts for about 65% of the total N available in the biosphere [49]. Inorganic nitrogen sources, such as nitrate (NO_3_^−^) and ammonium (NH_4_^+^), can be assimilated by several enzyme systems in order to provide the amino acids that are required for the synthesis of proteins and other essential compounds in plants [49,50]. In addition, organic N such as urea, can be transported into cells, its hydrolysis generates CO_2_ and ammonia, which is the only N form coming from urea that is accessible for assimilation. Later, ammonia can be converted in amino acids in the plastids [51,52,53]. In nature, the main sources of urea are urine excretion by animals, such as amphibians, and the decomposition of nitrogenous compounds from dead animals [52].

Ammonium and nitrate uptake into roots through soil is actively mediated by plasma membrane-localized transporters. For ammonium uptake, members of the ammonium transporter (AMT) family have been identified [54]. For nitrate, two transporter families have been identified: nitrate transporter 1 (NRT1)/peptide transporter (PTR) FAMILY (NPF) (previously named NRT1) and NRT2 [55,56]. Most of the NPF members are classified as low-affinity nitrate transporters, whereas NRT2 members are classified as high-affinity nitrate transporters. Urea can be transported into plant cells through a high-affinity active urea transporter as well as a passive transporter of urea. DUR3 is a high-affinity urea transporter and was previously identified in several species (e.g., *A. thaliana*, *Zea mays*, *Oryza sativa*) [52,57]. Passive urea transport is mediated by aquaporins. Among the subclasses of aquaporins, tonoplast intrinsic proteins (TIP), and Nodulin 26-like membrane intrinsic proteins (NIP) are known to transport urea in plants [58]. After being transported into the cells, inorganic and organic nitrogen sources need to be assimilated into amino acids through several enzyme systems. 

### 2.2. Nitrate, Ammonium, and Urea Assimilation

**Figure 2:**
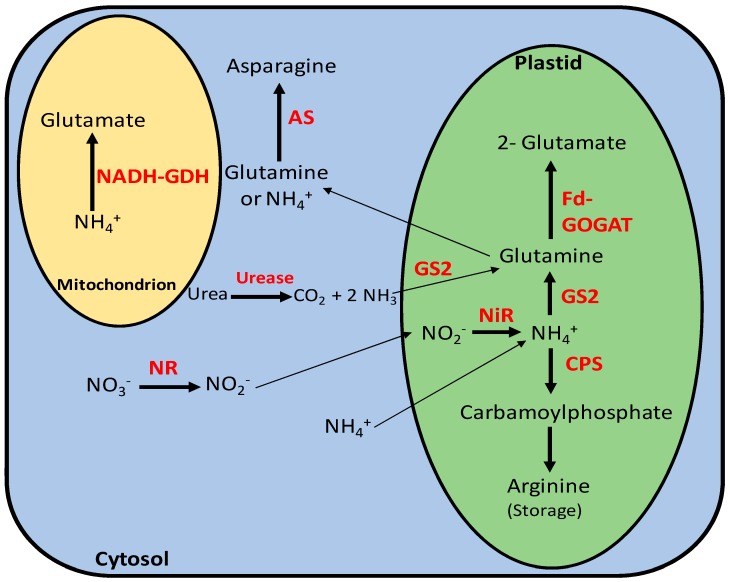
Schematic representation showing the main enzymes participating in NO_3_^−^, NH_4_^+^, and urea assimilation in plants. NR: nitrate reductase; NiR: nitrite reductase; GS2: glutamine synthetase 2; CPS: carbamoyl phosphate synthetase; Fd-GOGAT: glutamine oxoglutarate aminotransferase; AS: asparagine synthetase; NADH-GDH: glutamate dehydrogenase.

After being absorbed by the plants, nitrate is reduced to nitrite by the nitrate reductase (NR) enzyme in the cytosol of both roots and shoots by using ferredoxin (Fdx) or NADH as reducing power (Figure 2). Nitrite is known as a highly reactive compound and needs to be rapidly transported from the cytosol into the chloroplasts in the leaves and plastids in the roots. In these organelles, nitrite is reduced to NH_4_^+^ by the nitrite reductase enzyme (NiR) that uses NADH or Fdx as reducing power [49,57,58,59,60,61]. Ammonium originating from nitrite reduction, cytosolic ammonium, ammonium from photorespiration, amino acid recycling, and ammonia originating from cytosolic urea hydrolysis by urease, a nickel-dependent enzyme, are assimilated into the plastid by the glutamine synthetase enzyme (GS) to form the amino acid glutamine in an ATP-dependent reaction (Figure 2). There are two isoforms of the GS enzyme, GS1 and GS2. GS1 is encoded by the *GLN1* gene and it is located in the cytosol in *A. thaliana*. This isoform seems to be involved in the primary assimilation of ammonium from nitrate reduction and the assimilation of ammonium from photorespiration. GS2 is encoded by the *GLN2* gene in *A. thaliana* and is located in the chloroplast. This isoform seems to be involved in ammonium assimilation from amino acid recycling [60,62]. Glutamine reacts with 2-oxoglutarate and forms two molecules of glutamate by the action of the glutamate synthase enzyme (GOGAT) (Figure 2) [63,64]. In relation to glutamate synthase, two isoforms were also identified in plants: Fd-GOGAT and NADH-GOGAT. Fd-GOGAT is located in chloroplasts of photosynthetic tissues, whereas NADH-GOGAT is located in nonphotosynthetic tissues, such as companion cells and roots (Figure 2) [64,65].

Besides the GS/GOGAT isoforms, other enzymes also participate in ammonium assimilation. In the cytosol, asparagine synthetase (AS) is responsible for generating asparagine from glutamine or NH_4_^+^ in an ATP-dependent reaction (Figure 2) [66,67]. Three genes were identified in *A. thaliana* as being responsible for encoding the AS enzyme: *ASN1*, *ASN2*, and *ASN3* [64]. The carbamoyl phosphate synthase (CPS) enzyme produces carbamoyl phosphate by using NH_4_^+^ as its substrate in an ATP-dependent reaction that occurs within the chloroplast. Carbamoyl phosphate is the precursor of the amino acid arginine and can be stored in the plastid (Figure 2) [64]. The genes *carA* and *carB* were identified in *A. thaliana* and they are responsible for encoding the small and large subunits of the CPS enzyme [68]. In the mitochondrion, under high concentrations of ammonium, the NADH-glutamate dehydrogenase enzyme (NADH-GDH) can also convert NH_4_^+^ into glutamate [69].

### 2.3. Nitrate Effects on CAM

The influence of NO_3_^−^ on CAM expression has been documented in several obligate and facultative CAM species [43,45,70,71,72]. Researchers observed that *K. blossfeldiana* plants grown under 1 mM NO_3_^−^ or NH_4_^+^ despite having the same growth rates, showed higher nocturnal CO_2_ uptake and nocturnal malate content in the presence of NO_3_^−^ [70]. These observations indicate that under a higher concentration (10 mM), plants grown in the presence of ammonium developed necrosis at the base of the stem, indicating that higher concentrations of this N source were toxic for *K. blossfeldiana* [70], which was not observed in the presence of NO_3_^−^. Another study performed with this same species showed that there was a three-fold increase in the malate content after two months, and a five-fold increase in malate content after four months in the presence of 10 mM NO_3_^−^ compared to 10 mM NH_4_^+^ [73]. In addition, nocturnal CO_2_ uptake as well as the PEPC enzyme, phosphofructokinase (PFK), and glyceraldehyde-3-phosphate (G3PDH) activities were higher in the presence of nitrate compared to NH_4_^+^ [73]. Ota and Yamamoto [74] also observed in *Kalanchoë daigremontiana* three-fold lower nocturnal CO_2_ uptake in the presence of NH_4_^+^ compared with NO_3_^−^. Two obligate CAM species, *Kalanchoë laxiflora* and *Kalanchoë delagoensis*, grown under different concentrations of ammonium and/or nitrate showed a significantly higher nocturnal malate accumulation, ATP- and PPi-dependent proton transport into the vacuole in the presence 2.5 mM nitrate than other treatments [71]. As observed in other species, *K. laxiflora* and *K. delagoensis* decrease their total nocturnal organic acid accumulation (i.e., malate + fumarate + citrate) when grown in the presence of high concentrations of nitrate (5.0 mm). Nitrate is known to inhibit tonoplast V-ATPase activity, which together with V-PPiase is responsible for transporting protons and organic acids into the vacuole [75]. The inhibitory effect on V-ATPase activity, which is responsible for most of the protons transported into the vacuole at night in *Kalanchoë spp.*, caused by high concentrations of nitrate, might be responsible for decreasing the magnitude of CAM in both species [71].

In contrast to *Kalanchoë spp.*, when *G. monostachia* was grown in the presence of 5.0 mM NO_3_^−^ and exposed to water-deficit stress, the magnitude of CAM decreased by lowering nocturnal malate and citrate accumulation, PEPC and MDH activities, *GmoALMT9* expression (aluminum-dependent malate transporter), and the rates of ATP and PPi-dependent proton transport when compared to plants grown in the presence of 5.0 mM NH_4_^+^ and water-deficit stress. When lower concentrations of NO_3_^−^ were evaluated (e.g., 1.25 mM and 2.5 mM), a slight increase in PEPC activity was observed; however, it was still lower than when compared to the same concentrations of NH_4_^+^ [72]. Thus, the preference for each N source seems to depend upon the plant species, their habitat, and the relative toxicity of the N source for the plant. For bromeliads, higher concentrations of ammonium do not seem to be toxic and for some species it helps to increase CAM expression [72,76]. On the other hand, the preference for NO_3_^−^ over NH_4_^+^ for *Kalanchoë spp.* might be associated with the sensitivity of these plants to ammonium as well as the possible link between malate synthesis in the leaves and the assimilation of nitrate [43]. Malic acid has been proposed to be synthesized to neutralize the hydroxyl ions that are formed during the reduction of nitrate to ammonia, and this synthesis is thought to balance the residual inorganic cations [43,77]. Despite the many studies that have shown the influence of NO_3_^−^ on CAM, there is still a lack of information regarding how this N source influences CAM activity in different species.

Another subject that needs to be addressed is the relationship between nitrate and cytokinins, a hormone reported to be a negative regulator of CAM photosynthesis. Schmitt and Piepenbrock [78] reported that 6-benzylaminopurine (25 μM, 100 μM, and 400 μM) treatment resulted in a decline in relative PEPC transcript abundance, PEPC activity, and nocturnal organic acid accumulation in *M. crystallinum* leaves. In another C_3_-CAM species, *G. monostachia*, researchers observed a negative correlation between endogenous free cytokinins and PEPC activity [14]. Under water-deficit stress conditions, the concentrations of endogenous cytokinins decreased in the middle of the dark period, when the highest PEPC activity was observed. Nitrate is known for inducing *IPT* gene expression, which encodes the enzyme isopentenyltransferase, which participates in cytokinin biosynthesis [79]. Based on these observations, NO_3_^−^ could negatively regulate CAM induction by increasing cytokinin concentrations, which is a negative regulator of PEPC transcript accumulation and activity in *M. crystallinum* and *G. monostachia*. However, all *Kalanchoë spp.* used in CAM studies showed higher CAM expression in the presence of nitrate rather than ammonium. One possibility could be that NO_3_^−^ might negatively regulate CAM photosynthesis by increasing cytokinins in some facultative CAM species, but not obligate CAM species, or this regulation could just be specific to some species, such as *M. crystallinum* and *G. monostachia*, but not *Kalanchoë spp*. However, more studies need to be performed in order to better understand the relationship among CAM, NO_3_^−^, and cytokinin levels in plants. An alternative for this survey would be to maintain obligate and facultative CAM plants under different NO_3_^−^ concentrations in order to quantify endogenous cytokinin concentrations and the magnitude of CAM expression or CAM induction. Thus, it would be possible to verify which NO_3_^−^ concentration would positively regulate cytokinin concentrations and result in the largest degree in CAM expression.

### 2.4. Ammonium Effects on CAM

Ammonium concentrations in the soil depend upon several factors such as pH, temperature, chemical components of the soil, oxygenation, light, and CO_2_, among others [80,81,82]. Soils with low pH (pH < 5.5) and anoxic conditions are richer in NH_4_^+^ compared to NO_3_^−^ [83,84]. From the soil, ammonium can be incorporated into amino acids in the plastids or can be stored in the vacuole, which has both a lower pH and a higher ammonium concentration when compared to the cytosol [85]. Many ammonium-responsive genes are specifically regulated by shifts in extracellular pH. Thus, a specific ammonium signaling pathway exists based upon changes in pH associated with ammonium uptake [86]. When ammonium enters cells in greater quantities than nitrate, in rice for example, an alkalinization in the cytoplasm occurs, which enhances proton-coupled nitrate transport for cytosolic pH balance and results in a synergism of ammonium and nitrate uptake [86]. The passive transport of ammonia to the vacuole is mediated by aquaporins, specifically tonoplast intrinsic proteins (TIPs) [87,88]. In the vacuole, ammonia is protonated to form ammonium and this form is stored in the vacuolar lumen. The compartmentalization of ammonium into the vacuoles could be a strategy to avoid ammonium toxicity in more sensitive tissues/organelles of the plant [89]. When present in high concentrations in the tissue, ammonium can be toxic and cause several types of damage to plants, such as chloroplast ultrastructure harm, depletion of carbon supply, interference with hormonal homeostasis and photosynthesis, excessive energy demands, increases in proton efflux, oxidative stress, shifts in cellular pH, and deficiency of mineral cations [90,91,92,93]. These toxicity responses to ammonium seem to be dependent upon the plant species. Studies have shown that rice and some bromeliad species are more tolerant to ammonium [72,94,95,96]. As mentioned earlier, in *G. monostachia*, the presence of ammonium (and absence of nitrate) plus water-deficit stress increased CAM activity by increasing relative water content, soluble sugar content, and antioxidant enzyme activities. In this facultative CAM species, ammonium seems to increase the plant’s tolerance to water-deficit stress, promoting an osmotic adjustment as well as limiting the oxidative damage. Consequently, in the presence of ammonium, *G. monostachia* exhibited stronger CAM expression, characterized by higher malate and citrate accumulation, as well as higher PEPC and MDH activities, when compared to the presence of nitrate [72]. When the leaves were kept under different concentrations of ammonium or nitrate (e.g., 1.25 mM, 2.5 mM, and 5.0 mM), PEPC activity increased as ammonium concentration increased, whereas in the presence of different concentrations of nitrate, PEPC activity did not show any significant difference [72]. *Moricandia arvensis*, a C_3_-C_4_ species, presented a higher malate content and a lower CO_2_ compensation point under NO_3_^−^ rather than in the presence of NH_4_^+^ [97]. *Zea mays*, a C_4_ species kept under 4 mM and 12 mM NH_4_^+^, accumulated less biomass and showed photosynthetic rates of 87% and 82%, respectively, of those plants kept under the same NO_3_^−^ concentrations [98]. In general, as with most CAM and C_3_ plants, C_4_ species seem to exhibit a preference for nitrate over ammonium as was observed by the higher organic acid accumulation and photosynthetic rates under the presence of nitrate. However, studies investigating the influence of inorganic nitrogen sources on the induction of CAM photosynthesis in C_4_-CAM species, such as *Portulaca grandiflora* and *Portulaca oleracea*, are still missing. These reports would help us to better understand what the best conditions for plants are to switch from C_4_ to CAM. Such knowledge will be especially beneficial to know when bioengineering CAM plants, possibly into C_3_ and C_4_ crops, when environmental conditions will increasingly become a challenge for the survival and productivity of those C_3_ and C_4_ plants due to climate change.

Many studies have pointed to different reasons for ammonium toxicity, such as pH disturbances, NH_4_^+^ uptake associated with proton extrusion, and shifts in plant carbohydrates [99]. Britto et al. [100] proposed a new hypothesis for NH_4_^+^ toxicity, which is a result of the energetic cost of pumping NH_4_^+^ out of the cells when it is in high concentrations in sensitive species. In barley, ATP:NH_4_^+^ stoichiometry was close to 1.0, which means the efflux of one NH_4_^+^ is accompanied by the hydrolysis of one ATP or the influx of one H^+^ per NH_4_^+^ that is extruded, which also counts as the expense of one ATP [90,100]. In barley and likely other NH_4_^+^-sensitive species, the cost of transporting one cationic species showed a significant effect on the energy balance of the whole plant. These findings could be important in order to better understand the concentrations at which NH_4_^+^ could be toxic for CAM plants, such as *K. blossfeldiana*, but not for other CAM species, such as *G. monostachia*. Therefore, species that are nonsensitive to ammonium probably would not have the need to pump NH_4_^+^ out of the cells as much as would be necessary for sensitive species. Physiological, genomics, proteomics, and metabolomics analysis could help us understand the optimization of NH_4_^+^ sensitivity in CAM species, and importantly in CAM crops. Ortholog genes responsible for ammonium tolerance in some CAM plants could be identified, cloned, and then transferred to NH_4_^+^-sensitive CAM crops in order to increase nitrogen-use efficiency (NUE) by improving N acquisition in soils with low ammonium concentration [92,101]. CAM plants have lower levels of RUBISCO than C_3_ species, around 10–35% less, and because this enzyme is rich in nitrogen, CAM plants can have lower nitrogen concentrations, suggesting that CAM plants have higher nitrogen-use efficiencies when compared with C_3_ plants [2].

Despite the toxicity effects that ammonium showed for most CAM plants, including obligate and facultative CAM species, NH_4_^+^ can be advantageous for plant growth due to the low energy requirement needed for its assimilation, when compared to NO_3_^−^ and urea. While NO_3_^−^ and urea have to be reduced to ammonium to be incorporated into amino acids, NH_4_^+^ itself is readily available to be directly assimilated into amino acids (Figure 2). The energy saved by NH_4_^+^ assimilation under stressful environmental conditions, such as water-deficit stress, could reduce the consumption of carbohydrates and alleviate the reduction in biomass, as was previously observed in rice seedlings [102]. Although rice is a C_3_ species, the same mechanism and preference for ammonium over nitrate could be used for some CAM species, as was recently reported in *G. monostachia* [72]. In addition, other facultative or obligate CAM species might show this same preferential response for ammonium over nitrate, as this relationship between nitrogen sources and CAM photosynthesis continues to be investigated in more CAM species through future research.

### 2.5. Organic N Source Effects on CAM

The influence of organic N sources on CAM photosynthesis has not been thoroughly studied in CAM species. Almost all of the studies have focused on the influence of nitrate and/or ammonium, but not organic N sources such as urea and glutamine. A study conducted on *A. thaliana* plants, a C_3_ species, observed that the N absorbed as NO_3_^−^ was partitioned to a larger extent to the shoots, while the N absorbed as glutamine was partitioned to a larger extent to the roots [103]. A high value of plant root mass growing on poor soils might result from the presence of organic sources of N; conversely, high shoot mass might result from higher rates of NO_3_^−^ in the soils [103]. These studies could help to explain not only the main source of N present in the soil, but also how it could interfere with the whole plant metabolism and photosynthetic pathways. Britto and Kronzucker [104] showed that high glutamine concentrations can stimulate PEPC expression, probably in order to supply 2-oxo acids, drawn to the TCA cycle through amino acid synthesis. Based on these observations, the presence of glutamine as a source of N for CAM species could positively increase CAM photosynthesis, because PEPC might be stimulated by this organic source of N.

The passive transport of urea to the vacuole can be mediated by aquaporins, specifically the tonoplast intrinsic protein (TIP) [105]. In *A. thaliana*, *AtTIP2;1* facilitated urea transport; in addition, the authors suggested that TIPs might play a role in equilibrating urea concentrations in different cellular compartments in plants [105]. In natural conditions, urea derived from excretions of amphibians can be found as an organic source of N in the tank of bromeliad species, such as *Vriesea gigantea* [86]. In this bromeliad species, urease, the enzyme responsible for urea hydrolyses into NH_3_ and CO_2_, was, on average, 61% localized in the cytoplasm near the chloroplasts [106]. Because the hydrolyses of urea releases CO_2_, it could be hydrated to form HCO_3_ in mesophyll cells, which is used as a substrate by PEPC, or it could be fixed by RUBISCO depending upon the photosynthetic pathway. However, it remains unclear how CAM species would use CO_2_ from urea hydrolysis, and if they might prefer urea as a source of N over nitrate or ammonium. A recent report showed that the presence of urea and water-deficit stress resulted in lower PEPC and MDH activities in the leaves of *G. monostachia*, compared to the presence of nitrate or ammonium and water-deficit stress [41]. More studies need to be done in order to better understand how organic sources of nitrogen, such as glutamine, urea, and arginine, could contribute to increasing the magnitude and induction of CAM expression in plants, and how organic N could be more advantageous for CAM plants compared with NO_3_^−^ and NH_4_^+^ in terms of metabolic costs.

### 2.6. Nitrogen Deposition

Nitrogen deposition is described as the input of reactive nitrogen forms from the atmosphere to the biosphere as dry and wet deposition [107]. A recent study reported an increase of 8% in global inorganic nitrogen deposition from 1984 to 2016 [108]. Inorganic nitrogen deposition, from fossil fuel combustion and excess fertilizer application, has increased in East Asia and Southern Brazil while there has been a decrease in Europe [108]. Nitrogen deposition is the third largest cause of global biodiversity loss, followed by changes in land use and global climate change [109]. Under higher concentrations of N resulting from nitrogen deposition, plants that grow quickly will boost their growth compared to slower-growing species. The faster-growing species can shade and outcompete their neighbors, thereby decreasing local biodiversity [110]. In sensitive ecosystems, it seems that nitrogen deposition makes the negative effects of drought conditions even worse, because non-native species benefit more from these conditions than native species [111]. The increase of N availability in soil might reduce biomass allocation in roots, increase evapotranspiration, and decrease root:shoot ratios [112]. These effects may negatively interfere with water uptake and the drought tolerance of plants. In this way, nitrogen deposition could increase plant susceptibility to drought [113]. As previously discussed, CAM plants are stronger competitors than C_3_ species under water-deficit conditions independent of the N concentrations of the soil [46]. For this reason, not only under global climate changes, but also under higher nitrogen deposition over the next decades, CAM species might provide a useful alternative to alleviate the effects of drought, high temperatures, and high inorganic nitrogen deposition in soils by better assimilating CO_2_ under environmental conditions compared with C_3_ and C_4_ species. However, more surveys need to be performed in order to establish which biotic and/or abiotic condition would increase CAM expression and facilitate atmospheric CO_2_ sequestration by these species in order to decrease the negative effects of higher atmospheric CO_2_ over the next few decades.

## 3. Conclusions and Perspectives

Several studies performed on CAM plants have shown the importance of nutrient availability, mostly nitrogen, on CAM expression. Nitrogen deficiency (and specific N sources) was proven to be an important abiotic condition to increase the magnitude of CAM in *Guzmania monostachia*, a facultative CAM species, and several obligate CAM *Kalanchoë spp.*. Some CAM species exhibit a preference for ammonium over nitrate, while other CAM plants have shown the opposite preference. However, most of these studies do not explore preferences with regard to the metabolic, genomic, and proteomic factors of CAM photosynthesis. Some studies focus on how macronutrient availability affects the mRNA expression of core CAM genes in order to provide a better understanding of the relationship between CAM and nutrients. In addition, compared to C_3_ plants, CAM plants seem to have higher nitrogen-use efficiencies [2,114] because of the use of PEPC for nocturnal atmospheric CO_2_ assimilation instead of RUBISCO. However, more experimental evidence is needed to confirm this hypothesis. For these purposes, and in order to answer important questions regarding the relationship between CAM and nutrient availability, CAM plants such as *Ananas comosus* and *Kalanchoë fedtschenkoi* could be used for future experiments because their genomes have been sequenced recently. Core CAM genes from these species could be targeted in order to evaluate how they might influence NUE and also how different macronutrient availability might influence the expression of these core CAM genes. Furthermore, studies on the relationships between CAM and macronutrients, including not only N, but also K^+^, PO_4_^2−^, and Ca^2+^, would be beneficial to improve the growth and yield of CAM crops, to enhance the environmental productivity index in order to have more precise models for estimating the productivity of CAM plants, and to provide a better understanding of how macronutrient availability, associated with NUE and WUE, might help CAM plants respond and survive under global climate changes.
